# Association of *UGT1A1* gene variants, expression levels, and enzyme concentrations with 2,3,7,8-TCDD exposure in individuals exposed to Agent Orange/Dioxin

**DOI:** 10.1038/s41598-024-54004-0

**Published:** 2024-02-09

**Authors:** Ha Van Quang, Nguyen Ba Vuong, Bui Ngoc Linh Trang, Nguyen Linh Toan, Hoang Van Tong

**Affiliations:** 1 Department of Haematology, Toxicology, Radiation, and Occupation, 103 Military Hospital, Vietnam Military Medical University, Hanoi, Vietnam; 2 The Center of Toxicological and Radiological Training and Research, Vietnam Military Medical University, Hanoi, Vietnam; 3 Institute of Biomedicine and Pharmacy, Vietnam Military Medical University, 222 Phung Hung, Ha Dong, Hanoi, Vietnam; 4 Department of Pathophysiology, Vietnam Military Medical University, Hanoi, Vietnam

**Keywords:** Agent Orange/Dioxin, 2,3,7,8-TCDD, UGT1A1 enzyme, *UGT1A1* gene polymorphism, Risk factors, Gene expression, Genetic association study, Pollution remediation

## Abstract

Among the congener of dioxin, 2,3,7,8-TCDD is the most toxic, having a serious long-term impact on the environment and human health. UDP-glucuronosyltransferase 1A1 (UGT1A1) plays a crucial role in the detoxification and excretion of endogenous and exogenous lipophilic compounds, primarily in the liver and gastrointestinal tract. This study aimed to investigate the association of *UGT1A1* gene polymorphisms, expression levels, and enzyme concentration with Agent Orange/Dioxin exposure. The study included 100 individuals exposed to Agent Orange/Dioxin nearby Da Nang and Bien Hoa airports in Vietnam and 100 healthy controls. *UGT1A1* SNP rs10929303, rs1042640 and rs8330 were determined by Sanger sequencing, mRNA expression was quantified by RT-qPCR and plasma UGT1A1 concentrations were measured by ELISA. The results showed that *UGT1A1* polymorphisms at SNPs rs10929303, rs1042640 and rs8330 were associated with Agent Orange/Dioxin exposure (OR = 0.55,* P* = 0.018; OR = 0.55, *P* = 0.018 and OR = 0.57, *P* = 0.026, respectively). *UGT1A1* mRNA expression levels and enzyme concentration were significantly elevated in individuals exposed to Agent Orange/Dioxin compared to controls (*P* < 0.0001). Benchmark dose (BMD) analyses showed that chronic exposure to 2,3,7,8-TCDD contamination affects the *UGT1A1* mRNA and protein levels. Furthermore, UGT1A1 polymorphisms affected gene expression and enzyme concentrations in individuals exposed to Agent Orange/Dioxin. In conclusion, *UGT1A1* gene polymorphisms, *UGT1A* gene expression levels and UGT1A1 enzyme concentrations were associated with Agent Orange/Dioxin exposure. The metabolism of 2,3,7,8-TCDD may influence *UGT1A* gene expression and enzyme concentrations.

## Introduction

Between 1962 and 1971, the US forces sprayed approximately 46 L of Agent Orange, a 50/50 mixture of 2,4-dichlorophenoxyacetic acid and 2,4,5-trichlorophenoxyacetic acid, in South Vietnam. The spray contained 2,3,7,8-tetrachlorodibenzop-dioxin (2,3,7,8-TCDD), the most toxic congener of dioxin, and has had serious long-term impacts on the environment^1^. Reports indicate that levels of 2,3,7,8-TCDD in total Agent Orange ranged from 6.2 to 14.3 p.p.m, averaging 13.25 p.p.m^1^. Among the areas most heavily affected by Agent Orange/dioxin, Bien Hoa and Da Nang airfields are known to be the two most dioxin-contaminated areas in Vietnam, and are named Agent Orange hotspots^2–4^. Previous studies have shown that large amounts of 2,3,7,8-TCDD have been found in soil, aquatic sediments, food and human blood in these two areas^5–7^.

Dioxins are a class of structurally and chemically connected compounds that include polychlorinated dibenzo-p-dioxins (PCDD or dioxins) and polychlorinated dibenzofurans (PCDF or furans), collectively referred to as dioxins^8^. They are persistent organic pollutants that are primarily produced through industrial combustion processes such as waste burning, as well as the production of chlorophenols and chlorophenoxy herbicides. Additionally, dioxins can result from the processing of metals, bleaching of paper pulp with free chlorine, and the production and use of dioxin-like polychlorinated biphenyls^8^. Dioxin persists in the environment and enters the body through several routes, with the gastrointestinal tract being the main route, accounting for 90 percent of the intake. Once in the body, dioxin is distributed to other organs via the circulatory system. However, as it is insoluble in water, it only exists in the blood for a short time before accumulating in adipose tissue and the liver^2,9^. In the liver, dioxin is metabolized by an enzyme system to form non-toxic or less toxic products.

UDP-glucuronosyltransferase 1A1 (UGT1A1) is the sole enzyme responsible for the glucuronidation of bilirubin in humans^10^. It plays a crucial role in the detoxification and excretion of endogenous and exogenous lipophilic compounds, mainly in the liver and gastrointestinal tract^11–13^. The *UGT1A1* gene encodes 13 isoenzymes, of which nine (*UGT1A1, UGT1A3, UGT1A4, UGT1A5, UGT1A6, UGT1A7, UGT1A8, UGT1A9*, and *UGT1A10*) are functional and four (*UGT1A2P, UGT1A11P, UGT1A12P,* and *UGT1A13P*) are pseudogenes formed by genetic modification of the exon 1 region of this gene in humans^14,15^. The *UGT1A1* gene is located on chromosome 2q37 and employs alternative promoter and coding regions to produce the UGT1A1 enzyme. Most toxins, including dioxin, are metabolized and excreted in the liver. Previous studies have reported that *UGT1A1* genetic mutations and gene expression levels are associated with 2,3,7,8-TCDD^16–19^. Specifically, the UGT1A1 enzyme participates in the metabolism and elimination of xenobiotics, including dioxin, through the AhR pathway^20,21^.

Variations in the *UGT1A1* promoter and coding regions can influence *UGT1A* mRNA expression levels and *UGT1A* protein synthesis^17^, as well as its effect on the enzyme activity of UGT1A1. This enzyme is responsible for illnesses such as Gilbert syndrome (GS), Crigler-Najjar syndrome type I (CNI), and Crigler-Najjar syndrome type II (CNS), all of which cause unconjugated hyperbilirubinemia (CNII)^22–24^. However, studies on *UGT1A1* gene polymorphisms, gene expression levels, and enzyme concentrations in individuals exposed to Agent Orange/Dioxins have received little attention. Most recent studies have used experimental animals to evaluate the impact of dioxin on metabolic diseases. Therefore, this study aims to investigate the relationships between 2,3,7,8-TCDD and enzyme concentrations, *UGT1A1* gene expression levels, and polymorphisms in humans exposed to Agent Orange/Dioxins.

## Materials and methods

### Study areas

Bien Hoa and Da Nang Air Force Bases are considered to be major hotspots of dioxin contamination in Vietnam due to the large quantities of herbicides that were stockpiled in these areas during the war^3^. The highest concentration of dioxins ever measured at Da Nang Airport was 365,000 pg Toxicity Equivalent (TEQ)/g in soil samples collected from the former Agent Orange mixing and loading area^25^. A recent study has shown that the Toxic Equivalent Quotients (TEQs) for PCDD/F in soil and sediment samples from these areas ranged from 7.6 to 962,000 and 17 to 4860 pg/g dry weight, respectively^4^. The people who live near these areas face a high risk of dioxin exposure due to their proximity to areas heavily contaminated with Agent Orange/dioxin. Additionally, large amounts of 2,3,7,8-TCDD have been detected not only in individuals working at these airbases but also in nearby residents^5,7^. Therefore, we chose these two areas for our study.

### Study subjects

The study subjects consisted of 100 participants who had been exposed to Agent Orange/dioxin (dioxin group) and 100 healthy people (control group). All subjects are part of the same Vietnamese population, specifically the Kinh ethnicity with the same genetic background. These study subjects have been used and mentioned in a previous study^26^. Briefly, dioxin group selection criteria include: (i) live near an airport for at least five years; (ii) be 18 years or older and willing to participate in the study; and (iii) have a dioxin TEQ higher than 10 pg/g lipid in their serum and a 2,3,7,8-TCDD TEQ rate in total dioxin higher than 30 percent. The inclusion criteria aimed to select subjects who were contaminated with PCDDs and PCDFs used during the war. Age, gender, and the length of time spent in dioxin-contaminated areas at the time of recruitment were collected.

The control individuals who had no exposure to Agent Orange/dioxin were recruited between February and April 2021, at Vietnam Military Medical University. The inclusion criteria for control groups include: (i) willing to take part in the study; (ii) born and raised in Northern Vietnam; (iii) not part of the army during the war in South Vietnam; and (iv) not having metabolic disorders related to the UGT1A1 enzyme, such as cancer or hyperbilirubinemia. In addition, the age of the control group was similar to that of the dioxin group. The control group was carefully selected to ensure similarity in age with the dioxin group. The control individuals underwent comprehensive clinical examinations, including abdominal ultrasound, as well as tests for blood counts and various biochemical indicators. These biochemical indicators encompassed liver enzymes such as AST, ALT, and GGT, as well as levels of glucose, urea, creatinine, and bilirubin. Furthermore, markers for hepatitis B virus (HBV) infection were also assessed. Individuals presenting with anemia, elevated liver enzyme levels, hyperbilirubinemia, and specific diseases such as diabetes, HBV infection, and cancers were excluded from the study.

### Ethical approval

All study participants received thorough information about the investigation and informed consent was obtained from all participants. All methods were carried out in accordance with relevant guidelines and regulations such as clinical examination, sample and data collection and experimental procedures. The Vietnam Military Medical University Ethics Board evaluated and approved a procedure that was followed for all clinical procedures.

### Quantification of Agent Orange/Dioxin concentration

The concentrations of 2,3,7,8-TCDD were measured in the blood of all 100 study subjects exposed to Agent Orange/dioxin using liquid/solid extraction, followed by gas chromatography-high resolution mass spectrometry (GC/HRMS) analysis, in accordance with the U.S. Environmental Protection Agency (EPA) Method 1613 Rev.^27^. The detailed procedure has been described in a previous study^26^.

### Measurement of UGT1A1 enzyme concentration

Plasma samples were obtained by centrifuging 2 ml of blood collected from each participant and storing at − 80 °C until analysis. The Human UGT1A1 ELISA (Enzyme-Linked ImmunoSorbent Assay) Kit (MyBioSource, catalog number: MBS760703) was used to measure UGT1A1 concentration in plasma samples according to the manufacturer’s instructions. Briefly, the plasma samples were diluted fivefold with Sample Dilution Buffer and added to wells containing the standard solution, followed by incubation and washing steps. The diluted biotin-labelled antibody solution was added to each well, followed by incubation and washing steps. Then, horseradish peroxidase (HRP)-streptavidin conjugate was added and incubated at 37 °C for 30 min. The plate was washed five times and then incubated with tetramethylbenzidine (TMB) at 37 °C for 10–20 min. The reaction was stopped with the stop solution, and the optical density (OD) absorbance was read at 450 nm in a Microplate Reader immediately after adding the stop solution. The concentration of UGT1A1 enzyme in each sample was calculated based on the standard curve.

### Quantification of UGT1A1 gene expression levels

Total RNA was extracted from whole blood using the GeneJET RNA Purification Kit (Catalog Number: K0732, Thermo Fisher Scientific, USA). Reverse transcription into cDNA was performed using the RevertAid First Strand cDNA Synthesis Kit (Catalog Number: K1622), with cycling at 65 °C for 5 min, 42 °C for 60 min, and 70 °C for 5 min, followed by storage at − 80 °C until analysis. The *GAPDH* gene was used as an internal control^28^. The primer sequences used for *UGT1A1* gene expression were as follows: *UGT1A1* forward primer 5′-ATG CTG TGG AGT CCC AGG GC-3′ and UGT1A1 reverse primer 5′-CCA TTG ATC CCA AAG AGA AAA CC-3′; GAPDH forward primer 5′-GGT GGT CTC CTC TGA CTT CAA C-3′ and *GAPDH* reverse primer 5′-TCT CTC TTC CTC TTG TGT TCT TG-3′^29^. RT-qPCR was performed in a total volume of 20 µL, including 10 µL of SYBR Green PCR Master Mix 2X (Thermo Fisher Scientific, USA), 0.25 µM of each primer, 20 ng/µL of cDNA, and 6.5 µL of deionized water. A no-template control (H_2_O) was included in each reaction run. RT-qPCR for the *UGT1A1* gene was performed under the following conditions: an initial denaturation step of 10 min and 20 s at 95 °C, followed by 45 cycles of 95 °C for 20 s, 60 °C for 45 s, and final extension at 72 °C for 30 s. RT-qPCR for the *GAPDH* gene was then performed under the following conditions: 1 cycle at 95 °C for 5 min, followed by 45 cycles of 95 °C for 30 s, 63 °C for 45 s, and final extension at 72 °C for 30 s. The expression of the UGT1A1 gene was calculated using the 2^−ΔCt^ method, normalizing with the *GAPDH* gene^30^.

### Genotyping of UGT1A1 single nucleotide polymorphisms

Total DNA was extracted from whole blood samples using the Gene JET Whole Blood Genomic DNA Purification Mini Kit (ThermoFisher, USA; catalog number: K0782) following the manufacturer's instructions. A DNA fragment covering SNPs rs10929303 (1813C > T), rs1042640 (1941C > G) and rs8330 (2042C > G) of the *UGT1A1* gene in Exon5N2 region was amplified by PCR reaction using previously published primer pairs^31^, as follows: UGT1A1 forward primer: 5′–AAT TAA TCA GCC CCA GAG TGC–3′ and UGT1A1 reverse primer: 5′–GAA GGC GTG TGT GTG TGA AC–3′. The temperature cycling was as follows: 95 °C for 2 min, followed by 30 cycles of denaturation at 94 °C for 15 s, annealing at 54 °C for 30 s, and extension at 68 °C for 30 s, with a final extension for 5 min at 68 °C. The PCR products were purified using the GeneJet Genomic DNA Purification Kit (ThermoFisher, USA) and checked by agarose gel electrophoresis before subjecting to Sanger sequencing. The genotypes of *UGT1A1* SNPs rs10929303, rs1042640, and rs8330 in the target sequences were determined using the Bioedit software and deviations from Hardy–Weinberg (HW) equilibrium were tested. *UGT1A1* haplotypes were reconstructed based on three study SNPs using the expectation-maximum (EM) algorithm implemented in the Arlequin v. 3.5.1.2 software.

### Statistical analysis

The concentrations of 2,3,7,8-TCDD and UGT1A1 enzyme, and *UGT1A1* mRNA expression levels were tested for normality and presented as mean with standard deviation or medians with ranges. These data were also logarithmic transformed for further analyses. To compare the means or medians of continuous variables, Student’s t-test, ANOVA, Mann–Whitney U, or Kruskal–Wallis tests were used as appropriate. A linear regression model (univariate and multivariate) was employed to examine the correlation between variables. Particularly, the Benchmark dose (BMD) model was applied to evaluate the impact of chronic exposure to 2,3,7,8-TCDD contamination on the *UGT1A1* mRNA expression and UGT1A1 protein concentrations using PROAST version 70.1. We used the Akaike information criterion (AIC) model selection to select the best-fitted models for describing the relationship between variables. The genotype and allele frequencies, as well as the haplotype of the *UGT1A1*, were compared between groups using Chi-square or Fisher's exact tests. Odds ratios (ORs) and their 95% confidence intervals (CIs) were determined. Arlequin 3.1 and SPSS v.25 (SPSS Statistics, IBM, Armonk, NY, USA) were used for all statistical analyses. Statistical significance was defined as two-sided *P* values below 0.05.

## Results

### The characteristics and dioxin levels of study subjects

There was no significant difference between the mean age of the individuals exposed to Agent Orange/Dioxin (mean: 53.6 ± 10.1) and that of the healthy controls (mean: 53.0 ± 13.1). The median of blood 2,3,7,8-TCDD concentration was 43.74 pg/g lipid. The exposure time to Agent Orange/dioxin was 33 years^26^. We observed a positive correlation between the blood concentrations of 2,3,7,8-TCDD in individuals exposed to Agent Orange/Dioxin and their duration of exposure to Agent Orange/Dioxin (r = 0.436, *P* < 0.0001) (Suppl. Figure 1). However, the 2,3,7,8-TCDD concentrations were not determined for the control group.

### Associations of UGT1A1 gene polymorphisms with Agent Orange/Dioxin exposure

The distribution of allele and genotype frequencies of *UGT1A1* gene polymorphisms rs10929303, rs1042640, and rs8330 are presented in Table 1. The results indicate that the genotype frequencies of *UGT1A1* gene polymorphisms rs10929303, rs1042640, and rs8330 in the dioxin and control groups were in Hardy–Weinberg equilibrium (*P* > 0.05). The frequencies of minor alleles *rs10929303T, rs1042640G,* and *rs8330G* were significantly lower in individuals exposed to Agent Orange/Dioxin compared to healthy controls, suggesting that these alleles were associated with protection from Dioxin exposure (rs10929303T: OR = 0.55, 95%CI = 0.33–0.91, *P* = 0.018; rs1042640G: OR = 0.55, 95%CI = 0.33–0.91, *P* = 0.018; rs8330G: OR = 0.57, 95%CI = 0.35–0.94, *P* = 0.026) (Table 1).

**Table 1:** Genotype and allele distribution of *UGT1A1* gene polymorphisms in study groups.

*UGT1A1* SNP	Dioxin group n = 100 (%)	Control group n = 100 (%)	OR (95% CI)	*P* value
rs10929303C/T				
*CC*	72 (72)	58 (58)	Reference	NA
*CT*	25 (25)	34 (34)	0.59 (0.32–1.10)	0.097
*TT*	3 (3)	8 (8)	0.3 (0.08–1.19)	0.073
*C*	169 (84.5)	150 (75)	**0.55 (0.33–0.91)**	**0.018**
*T*	31 (15.5)	50 (25)		
rs1042640C/G				
*CC*	73 (73)	58 (58)	Reference	NA
*CG*	23 (23)	34 (34)	0.54 (0.29–1.01)	0.053
*GG*	4 (4)	8 (8)	0.4 (0.11–1.39)	0.136
*C*	169 (84.5)	150 (75)	**0.55 (0.33–0.91)**	**0.018**
*G*	31 (15.5)	50 (25)		
rs8330C/G				
*CC*	72 (72)	58 (58)	Reference	NA
*CG*	24 (24)	34 (34)	0.57 (0.30–1.06)	0.076
*GG*	4 (4)	8 (8)	0.4 (0.12–1.41)	0.143
*C*	168 (84)	150 (75)	**0.57 (0.35–0.94)**	**0.026**
*G*	32 (16)	50 (25)		

*P* values were calculated by using Chi-square tests. Bold ORs and *P* values indicate statistical significance.

We also reconstructed *UGT1A1* haplotypes based on the three study SNPs, observing two major haplotypes: *CCC* and *TGG*. The frequency of haplotype *TGG* was significantly lower in individuals exposed to Agent Orange/Dioxin than in the control group, indicating an association with protection from Dioxin exposure (OR = 0.54, 95%CI = 0.32–0.89, P = 0.014). Additionally, we observed two minor haplotypes, *CGG* and *TCG*, only in individuals exposed to Agent Orange/Dioxin (Table 2).

**Table 2:** Haplotype distribution of *UGT1A1* gene polymorphisms rs10929303, rs1042640 and rs8330 in study groups.

Haplotype	Dioxin n (%)	Controls n(%)	OR (95% CI)	*P* value
*CCC*	168 (84)	150 (75)	Reference	NA
*TGG*	30 (15)	50 (25)	**0.54 (0.32–0.89)**	**0.014**
*CGG*	1 (0.5)	0	NA	NA
*TCG*	1 (0.5)	0	NA	NA
Total	200	200		

*P* values were calculated by using Chi-square tests. Bold ORs and *P* values indicate statistical significance.

### UGT1A1 mRNA expression and Agent Orange/Dioxin exposure

**Figure 1:**
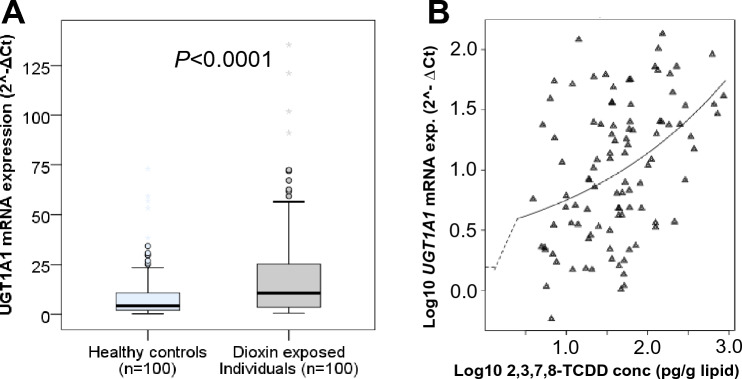
*UGT1A1* gene expression levels and its association with dioxin exposure. (**A**) *UGT1A1* gene expression levels in individuals exposed to Agent Orange/Dioxin and in healthy controls. *P* values were calculated by using the Mann–Whitney U test. (**B**) The effect of 2,3,7,8-TCDD exposure on *UGT1A1* mRNA expression levels was assessed by using Benchmark dose (BMD) model.

The *UGT1A1* mRNA expression levels in the blood samples of the study participants were analyzed and compared between individuals exposed to Agent Orange/Dioxin and healthy controls (Fig. 1A). The results indicated a significant increase in *UGT1A1* mRNA expression levels in individuals exposed to Agent Orange/Dioxin compared to healthy controls (*P* < 0.0001). A positive correlation was observed between *UGT1A1* mRNA expression levels and 2,3,7,8-TCDD concentrations in Agent Orange/Dioxin-exposed individuals (Spearman’s rho = 0.405, *P* < 0.0001). The benchmark dose (BMD) analyses showed that chronic exposure to 2,3,7,8-TCDD contamination affects the *UGT1A1* mRNA expression levels (Fig. 1B). We incorporated the UGT1A1 mRNA expression levels and UGT1A1 enzyme concentrations into a linear regression model to examine their correlation with the concentrations of 2,3,7,8-TCDD in individuals exposed to Agent Orange/Dioxin. The findings revealed a positive correlation between UGT1A1 mRNA expression levels and 2,3,7,8-TCDD concentrations (β = 1.204; *P* = 0.019).

### UGT1A1 enzyme concentration and Agent Orange/Dioxin exposure

**Figure 2:**
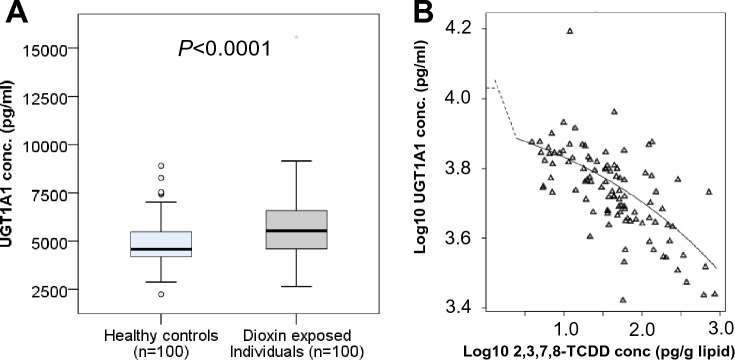
Enzyme UGT1A1 concentrations and their association with dioxin exposure. (**A**) Enzyme *UGT1A1* concentrations in individuals exposed to Agent Orange/Dioxin and in healthy controls. *P* values were calculated by using the Mann–Whitney U test. (**B**) The effect of 2,3,7,8-TCDD exposure on UGT1A1 concentrations was assessed by using Benchmark dose (BMD) model.

The UGT1A1 enzyme concentrations in the blood samples of study participants were analyzed and compared between Agent Orange/Dioxin-exposed individuals and healthy controls. The results revealed that UGT1A1 enzyme concentrations were significantly higher in Agent Orange/Dioxin-exposed individuals (median: 5507 pg/mL) compared to healthy controls (median: 4580 pg/mL) (*P* < 0.0001) (Fig. 2A). We analyzed the correlation of UGT1A1 enzyme levels with 2,3,7,8-TCDD levels in Agent Orange/Dioxin-exposed subjects. We observed an inverse correlation between the UGT1A1 enzyme and 2,3,7,8-TCDD concentrations (Spearman’s rho = − 0.678, *P* < 0.0001). The benchmark dose (BMD) analyses showed that chronic exposure to 2,3,7,8-TCDD contamination affects the UGT1A1 protein concentrations (Fig. 2B). Similarly, multivariate linear regression analysis showed that the UGT1A1 enzyme concentrations were inversely correlated with the 2,3,7,8-TCDD concentrations (β = − 0.036; *P* < 0.0001).

### Associations of UGT1A1 gene polymorphisms with plasma 2,3,7,8-TCDD concentrations

**Figure 3:**
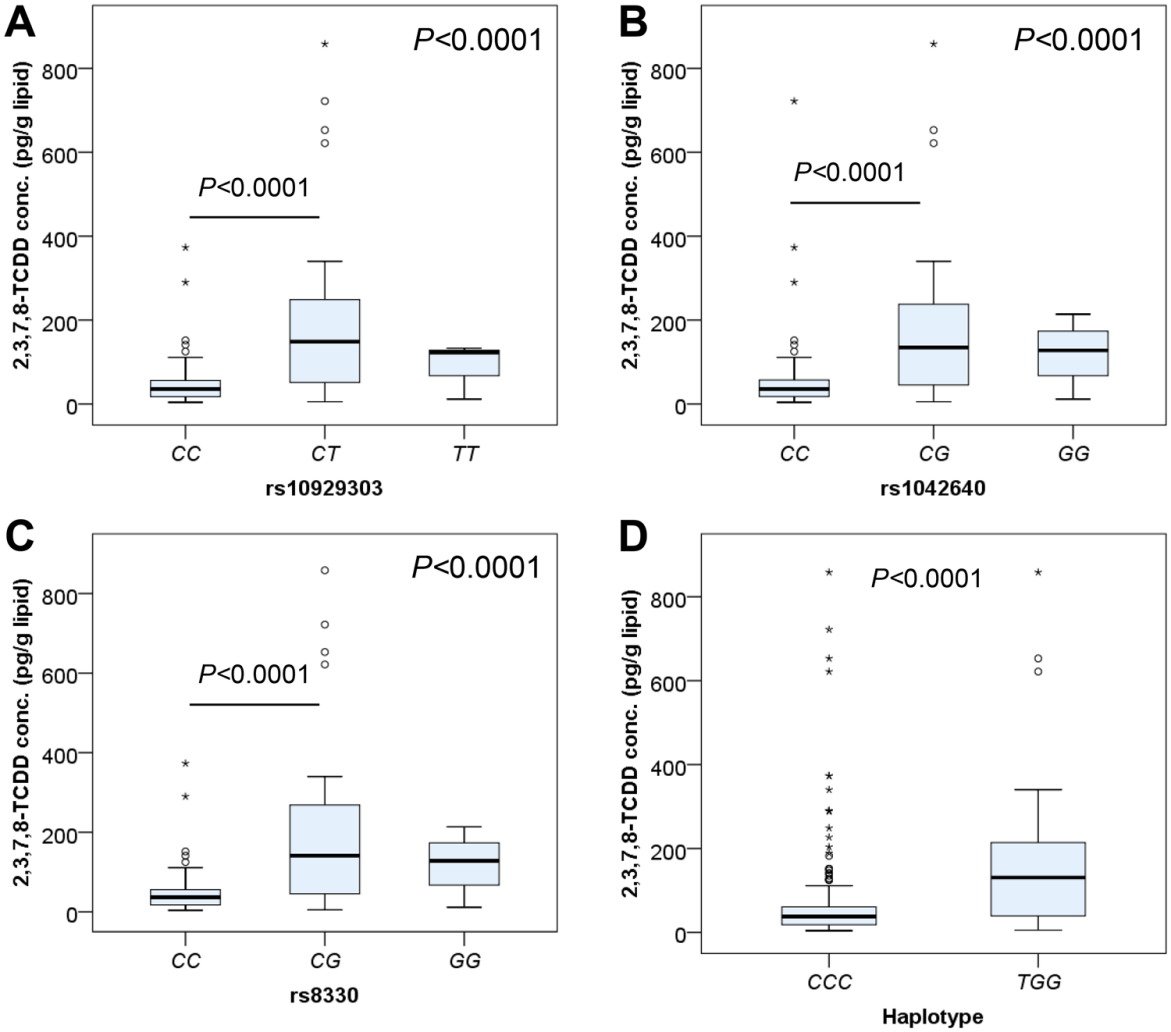
2,3,7,8-TCDD concentrations in Agent Orange/Dioxin exposed individuals with different *UGT1A1* genotypes. 2,3,7,8-TCDD concentrations in subgroups with different genotypes of the UGT1A1 SNPs rs10929303 (**A**), rs104260 (**B**) and rs8330 (**C**) and in subgroups with haplotype *CCC* and *TGG* based on three *UGT1A1* SNPs rs10929303, rs1042640 and rs8330 in individuals exposed to Agent Orange/Dioxin and in healthy controls. *P* values were calculated by using the Kruskal–Wallis tests.

We examined the relationship between *UGT1A1* gene polymorphisms and 2,3,7,8-TCDD concentrations in individuals exposed to Agent Orange/Dioxin. Based on their genotypes of *UGT1A1* polymorphisms rs10929303, rs1042640, and rs8330, Agent Orange/Dioxin-exposed individuals were divided into subgroups. With SNP rs10929303, individuals with the *CT* genotype had the highest 2,3,7,8-TCDD concentrations (median = 148.15 pg/g lipid), followed by individuals with the *TT* genotype (median = 123 pg/g lipid) and those with the *CC* genotype (median = 36.13 pg/g lipid) (*P* < 0.0001). With SNP rs1042640, individuals with the *CG* genotype had the highest 2,3,7,8-TCDD concentrations (median = 135 pg/g lipid), followed by individuals with the *GG* genotype (median = 128 pg/g lipid) and those with the *CC* genotype (median = 36.15 pg/g lipid) (*P* < 0.001). With SNP rs8330, individuals with the *CG* genotype had the highest 2,3,7,8-TCDD concentrations (median = 141.58 pg/g lipid), followed by individuals with the *GG* genotype (median = 128 pg/g lipid) and those with the *CC* genotype (median = 36.13 pg/g lipid) (*P* < 0.0001). Furthermore, individuals with the *TGG* haplotype (median = 130.7 pg/g lipid) had higher 2,3,7,8-TCDD concentrations than those with the *CCC* haplotype (median = 37.7 pg/g lipid) (*P* < 0.0001) (Fig. 3). These findings indicate that *UGT1A1* gene polymorphisms were significantly associated with 2,3,7,8-TCDD concentrations in Agent Orange/Dioxin-exposed individuals.

### UGT1A1 gene polymorphisms and the mRNA expression

**Figure 4:**
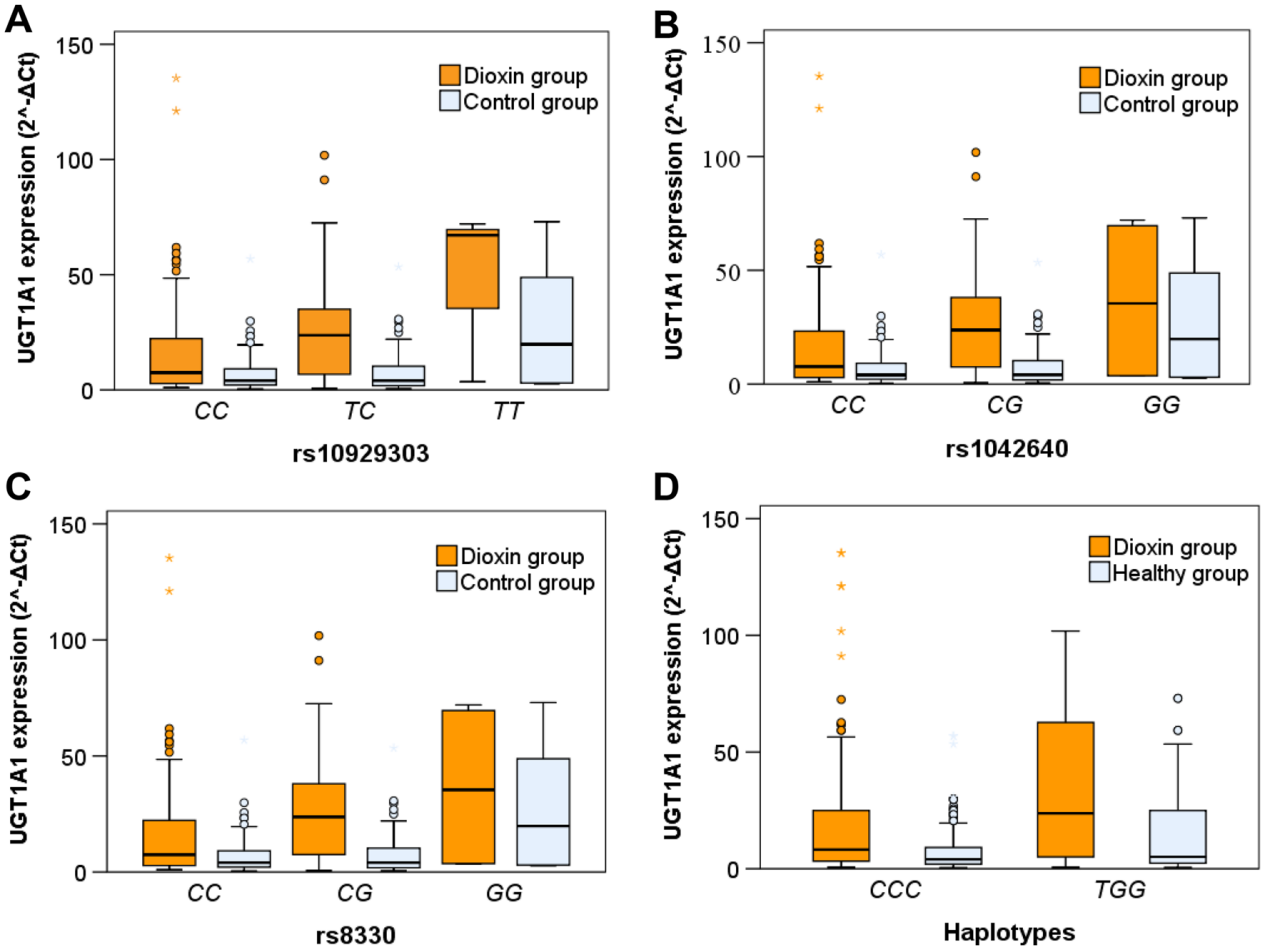
Association of *UGT1A1* SNPs and haplotype with gene expression in dioxin group and control group. *UGT1A1* mRNA expression levels in subgroups with different genotypes of the UGT1A1 SNPs rs10929303 (**A**), rs104260 (**B**) and rs8330 (**C**) and in subgroups with haplotype CCC and TGG based on three *UGT1A1* SNPs rs10929303, rs1042640 and rs8330 in individuals exposed to Agent Orange/Dioxin and in healthy controls. *P*-values of 0.02, 0.037, 0.023 and 0.007 were calculated for A, B, C and D, respectively, using the Kruskal–Wallis test for Dioxin group. For healthy group, *P*-values were > 0.05.

We analyzed the association between *UGT1A1* gene polymorphisms and its mRNA expression in Agent Orange/Dioxin-exposed individuals and healthy controls. In the dioxin group, individuals with genotypes *rs10929303TT, rs1042640GG,* and *rs8330GG* had the highest *UGT1A1* mRNA expression levels, followed by those with genotypes *rs10929303CT, rs1042640CG,* and *rs8330CG*, and those with genotypes *rs10929303CC, rs1042640CC,* and *rs8330CC*, respectively (*P* < 0.0001). Individuals with the *TGG* haplotype had significantly higher mRNA expression compared to those with the *CCC* haplotype (*P* = 0.007). Furthermore, we observed a similar trend in the control group, but the difference did not reach statistical significance (*P* > 0.05) (Fig. 4 and Supp. Table 1). These results indicate that the three SNPs, rs10929303, rs1042640, and rs8330, have an effect on *UGT1A1* gene expression, and alleles *rs10929303T, rs1042640G,* and *rs8330G* contribute to increased *UGT1A1* gene expression.

### UGT1A1 gene polymorphisms and enzyme concentrations

**Figure 5:**
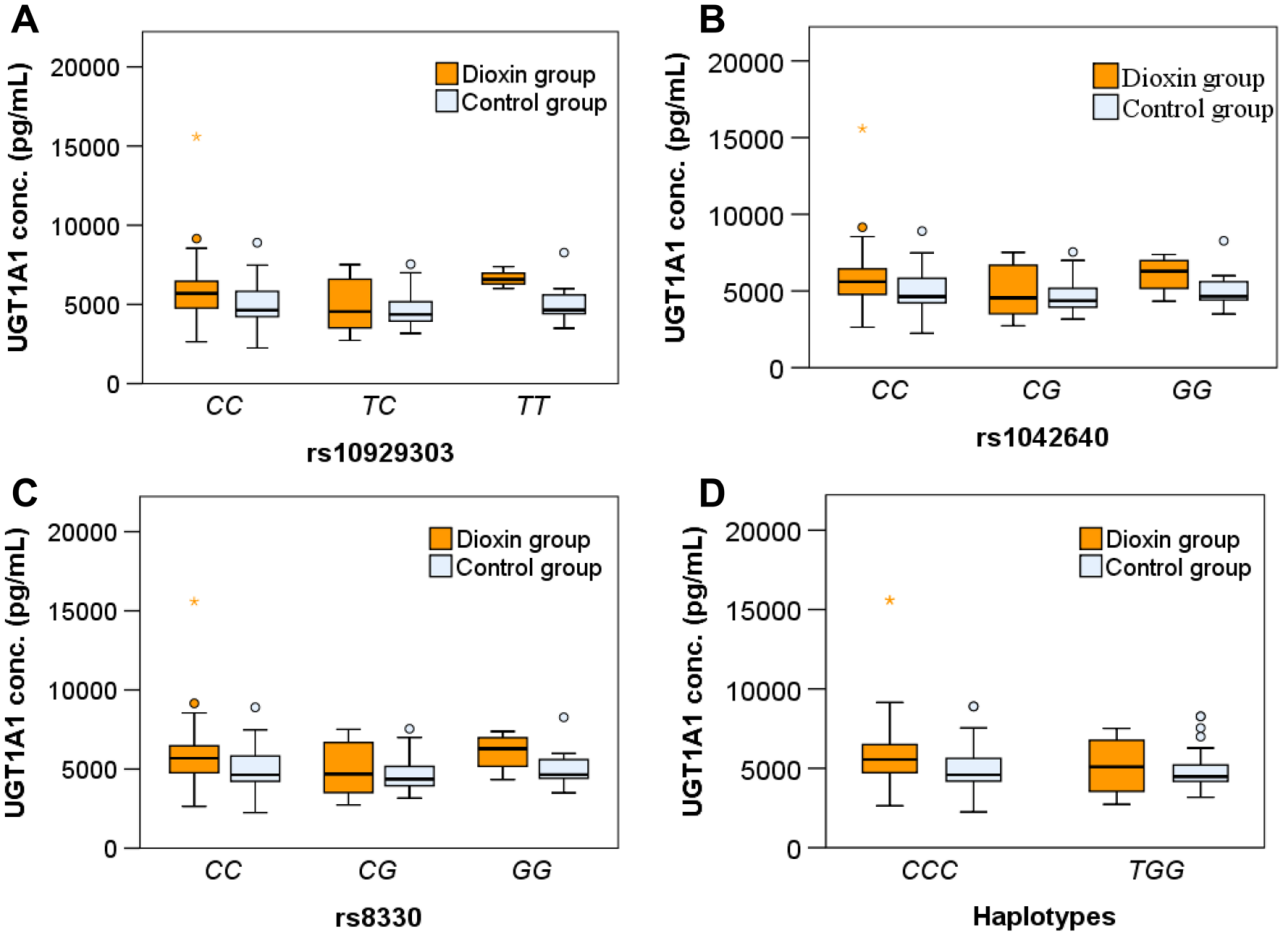
Association of *UGT1A1* SNPs and haplotype with UGT1A1 enzyme concentrations in the dioxin group and control group. Enzyme *UGT1A1* concentrations in subgroups with different genotypes of the UGT1A1 SNPs rs10929303 (**A**), rs104260 (**B**) and rs8330 (**C**) and in subgroups with haplotype CCC and TGG based on three *UGT1A1* SNPs rs10929303, rs1042640 and rs8330 in individuals exposed to Agent Orange/Dioxin and in healthy controls. *P*-values of 0.02, 0.073, 0.073 and 0.294 were calculated for A, B, C and D, respectively, using the Kruskal–Wallis test for Dioxin group. For healthy group, *P*-values were > 0.05.

We hypothesized that *UGT1A1* gene polymorphisms could influence plasma UGT1A1 enzyme concentrations. To examine this, we compared plasma UGT1A1 enzyme concentrations among individuals with different genotypes in two study groups. Among individuals exposed to Agent Orange/Dioxin, our results showed that individuals with genotypes *rs10929303TT, rs1042640GG,* and *rs8330GG* had the highest UGT1A1 enzyme concentrations, followed by those with genotypes *rs10929303CC, rs1042640CC,* and *rs8330CC*, and those with genotypes *rs10929303CT, rs1042640CG,* and *rs8330CG*, respectively (*P* < 0.0001). However, the difference in UGT1A1 enzyme concentrations among individuals with various genotypes was not significant in the control group (*P* > 0.05). Similarly, UGT1A1 enzyme concentrations were also not significantly different between individuals with haplotypes *CCC* and *TGG* in both the dioxin and control groups (*P* > 0.05) (Fig. 5 and Supp. table 1). These results suggest that *UGT1A1* gene polymorphisms may affect plasma UGT1A1 enzyme concentrations in individuals exposed to Agent Orange/Dioxin but not in healthy controls.

### UGT1A1 mRNA expression and UGT1A1 enzyme concentration

**Figure 6:**
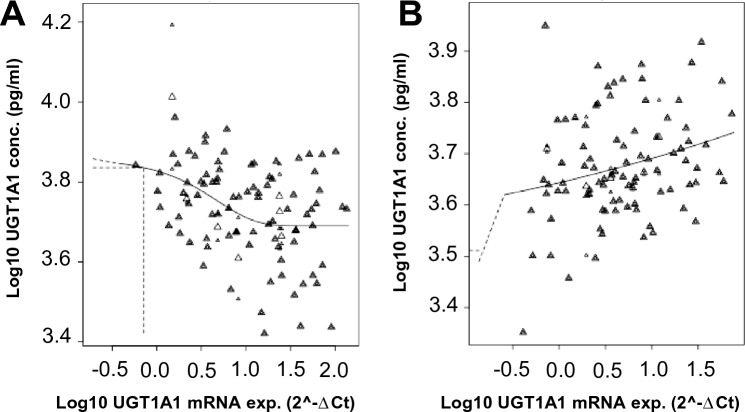
Correlations of *UGT1A1* gene expression levels with *UGT1A1* enzyme concentrations in the dioxin group and control group. (**A**) The correlation between *UGT1A1* gene expression levels and *UGT1A1* enzyme concentrations in individuals exposed to Agent Orange/Dioxin. The selected model is Expon. m5 (AICs of the Expon. m5 model is 26.58 vs. 36.14 of the null model). (**B**) The correlation between *UGT1A1* gene expression levels and *UGT1A1* enzyme concentrations in healthy controls. The selected model is Expon. m3 (AICs of the Expon. m3 model is − 2.14 vs. − 0.28 of the null model).

We performed a linear regression analysis to investigate the association between *UGT1A1* mRNA expression levels and UGT1A1 enzyme concentration. The results revealed an inverse correlation between *UGT1A1* mRNA expression levels and UGT1A1 enzyme concentration (Spearman’s rho = − 0.313, *P* < 0.001) in individuals exposed to Agent Orange/Dioxin. In contrast, a positive correlation was observed between *UGT1A1* mRNA expression levels and UGT1A1 enzyme concentrations in healthy controls (Spearman’s rho = 0.225, *P* = 0.024) (Fig. 6). These findings suggest that dioxin exposure has an impact on the plasma UGT1A1 enzyme concentrations in Agent Orange/Dioxin-exposed individuals.

## Discussion

In humans, TCDD is metabolized slowly and 2,3,7,8-TCDD is oxidized, chlorinated, and reduced by phase I metabolic enzymes, followed by a combined process by phase II enzymes, including the UGT1A1 enzyme^19,32^. *UGT1A1* gene polymorphisms are known to affect the body's response to xenobiotic exposure such as dioxin and drugs. To our knowledge, this is the first study to show the association between *UGT1A1* genetic variants and the status of exposure to Agent Orange/Dioxin containing 2,3,7,8-TCDD in the hotspots of dioxin contamination in Vietnam. The current study revealed that *UGT1A1* polymorphisms at SNPs rs10929303, rs1042640 and rs8330 were associated with the status of exposure to Agent Orange/Dioxin. *UGT1A1* mRNA expression levels and enzyme concentration were affected and correlated with 2,3,7,8-TCDD concentrations. The results also demonstrate the influence of *UGT1A1* polymorphisms on *UGT1A1* gene expression and enzyme concentrations in individuals exposed to Agent Orange/Dioxin.

The distribution of *UGT1A1* SNPs rs10929303 (1813C > T), rs1042640 (1941C > G) and rs8330 (2042C > G) genotypes and alleles in the Vietnamese population were similar to those reported in previous studies on Pakistani^11^ and Caucasian populations^33,34^. The *UGT1A1* homozygous variant (211G > A), but not the variants 1941C > G (rs1042640) and 2042C > G (rs8330), has been shown to be associated with increased bilirubin in Han Chines unconjugated hyperbilirubinemia neonates^35,36^. Similarly, a previous study reported that *UGT1A1* SNPs rs10929303, rs1042640 and rs8330 are associated with acetaminophen glucuronidation activity^34^. Another study found that *UGT1A1* SNP rs8330 is associated with the onset and prognosis of non-small cell lung cancer^37^. Our study reveals that *UGT1A1* polymorphisms at SNPs rs10929303, rs1042640 and rs8330 are associated with the status of exposure to Agent Orange/Dioxin. Furthermore, SNPs rs1092030 and rs8330 have been associated with different enzymatic activities, resulting in distinct glucuronidation phenotypes, including fast, intermediate, and slow metabolism. Specifically, individuals carrying the *rs1092030CC* and *rs8330CC* genotypes exhibit a fast glucuronidation phenotype, those with the *rs1092030CT* and *rs8330GT* genotypes display an intermediate glucuronidation phenotype, and individuals with the *rs1092030TT* or *rs8330GG* genotype demonstrate a slow glucuronidation phenotype^11^. We observed that individuals carrying the *rs1092030CC*, *rs1042640CC* and *rs8330CC* genotypes exhibited the lowest levels of 2,3,7,8-TCDD, indicating a fast glucuronidation phenotype. Conversely, those carrying the *rs1092030CT*, *rs1092030TT*, *rs1042640CG, rs1042640GG, rs8330CG*, and *rs8330GG* genotypes had higher levels of 2,3,7,8-TCDD, representing slower glucuronidation phenotypes. These findings suggest that *UGT1A1* gene polymorphisms (rs10929303, rs1042640 and rs8330) are factors significantly contributing to the metabolism of 2,3,7,8-TCDD.

Gene variation may influence gene expression, resulting in changes in the function and composition of essential pathway components, thereby influencing disease progression^38^. In particular, variants in the promoter and coding regions of *UGT1A1* can affect UGT1A mRNA expression levels and UGT1A1 protein synthesis^17^. A variant TATAA element in *UGT1A1* frequently causes decreased *UGT1A1* expression and enzyme activity, increasing the risk of clinical outcomes such as jaundice, present in both types 1 and 2 of Crigler-Najjar syndrome^39^. UGT1A1*28 variant is related to irinotecan-induced toxicities in Caucasian patients with colorectal cancer^40^. The *UGT1A1* SNP rs8330 is associated with gene expression, individuals with genotype *CC* exhibiting decreased expression of the *UGT1A1* gene compared to patients with genotypes *GG* and *CG*^37^. Consistent with previous observations, our findings indicate that *UGT1A1* SNPs rs10929303, rs1042640, and rs8330 are associated with increased levels of *UGT1A1* gene expression in individuals exposed to Agent Orange/Dioxin but not in controls. Therefore, *UGT1A1* gene polymorphisms (rs10929303, rs1042640, and rs8330) may be related to the metabolism of 2,3,7,8-TCDD, possibly by regulating *UGT1A1* gene expression. However, further studies are needed to clarify this association and the role of UGT1A1 enzymatic activity in 2,3,7,8-TCDD metabolism.

Variants in the *UGT1A1* exon or promoter regions can cause structural or functional defects in the protein, resulting in a decline in enzyme activity. Several studies have reported that SNPs in the *UGT1A1* gene can regulate UGT1A1 enzyme activity^24,41,42^. Additionally, *UGT1A1* gene variants can lead to a deficiency of UGT1A1 enzyme activity, as observed in Gilbert syndrome (GS) and Crigler-Najjar syndrome (CNS)^39^. Similarly, we found that *UGT1A1* SNP rs10929303 was related to increased UGT1A1 enzyme concentrations in the group exposed to 2,3,7,8-TCDD.

Our results indicate that exposure to dioxin had an effect on *UGT1A1* gene expression, leading to an elevation of the UGT1A1 enzyme concentration in dioxin-exposed groups compared to healthy controls. Particularly, dioxin exposure may promote *UGT1A1* mRNA expression and/or enzyme degradation while inhibiting protein synthesis and/or enzyme activity. Earlier studies also observed that 2,3,7,8-TCDD can induce *UGT1A*1 gene expression^19,43^ and that Agent Orange/Dioxin exposure can inhibit the activity of neuronal acetylcholinesterase (AChE) in an in vitro study^44^ and pyruvate kinase in a clinical observation^26^. Therefore, our findings suggest that *UGT1A1* gene polymorphisms (rs10929303, rs1042640, and rs8330) as well as exposure to 2,3,7,8-TCDD contribute to the regulation of *UGT1A1* gene expression.

Although this study is the first to examine *UGT1A1* gene polymorphism, gene expression, and enzyme concentration in individuals exposed to Agent Orange/Dioxin in Vietnam, it has several limitations. The first limitation is the lack of 2,3,7,8-TCDD concentration data in the control group. However, we carefully selected individuals who had never been exposed to Agent Orange/Dioxin or had any metabolic conditions linked to the UGT1A1 enzyme. A second limitation is the absence of UGT1A1 enzymatic activity data. As a result, we were unable to analyze the functional implications of different glucuronidation phenotypes (fast, intermediate, and slow) associated with *UGT1A1* SNPs in dioxin metabolism. Another limitation is the small sample size included for the case–control analysis, which prevented us from reaching statistical significance.

In conclusion, our findings indicate that Agent Orange/Dioxin exposure is associated with *UGT1A1* gene polymorphisms, *UGT1A* gene expression levels, and UGT1A1 enzyme concentrations. Exposure to 2,3,7,8-TCDD may influence *UGT1A* gene expression and enzyme concentrations. Our results suggest that exposure to 2,3,7,8-TCDD may impact human health by regulating *UGT1A1* gene expression and its functional activity. However, further studies are needed to clarify the role of *UGT1A1* gene variation and expression in dioxin metabolism.

### Supplementary Information

Supplementary Information.

## Data Availability

The datasets generated during the current study are available in the NCBI GenBank repository (Submission # 2794145).

## References

[1] Stellman JM, Stellman SD, Christian R, Weber T, Tomasallo C (2003). The extent and patterns of usage of Agent Orange and other herbicides in Vietnam. Nature.

[2] Schecter A (1995). Agent Orange and the Vietnamese: The persistence of elevated dioxin levels in human tissues. Am. J. Public Health.

[3] Dwernychuk LW (2005). Dioxin hot spots in Vietnam. Chemosphere (Oxford).

[4] Van Thuong N (2015). Transport and bioaccumulation of polychlorinated dibenzo-p-dioxins and dibenzofurans at the Bien Hoa Agent Orange hotspot in Vietnam. Environ. Sci. Pollut. Res. Int..

[5] Van Manh P (2021). Serum dioxin concentrations in military workers at three dioxin-contaminated airbases in Vietnam. Chemosphere.

[6] Minh NH, Tran TM, Hue NTM, Minh TB, Tuyet-Hanh TT (2019). Bioaccumulation of PCDD/Fs in foodstuffs near Bien Hoa and Da Nang airbases: Assessment on sources and distribution. Environ. Sci. Pollut. Res..

[7] Pham DT (2015). Predictors for dioxin accumulation in residents living in Da Nang and Bien Hoa, Vietnam, many years after Agent Orange use. Chemosphere.

[8] Schecter A, Birnbaum L, Ryan JJ, Constable J (2006). D. Dioxins: An overview. Environ Res.

[9] Djien Liem A, Furst P, Rappe C (2000). Exposure of populations to dioxins and related compounds. Food Addit. Contam..

[10] Liu W (2014). Genetic factors affecting gene transcription and catalytic activity of UDP-glucuronosyltransferases in human liver. Hum. Mol. Genet..

[11] Mehboob H (2017). Effect of UDP-glucuronosyltransferase (UGT) 1A polymorphism (rs8330 and rs10929303) on glucuronidation status of acetaminophen. Dose-Response.

[12] Lautala P, Ethell BT, Taskinen J, Burchell B (2000). The specificity of glucuronidation of entacapone and tolcapone by recombinant human UDP-glucuronosyltransferases. Drug Metab. Dispos..

[13] Watanabe Y, Nakajima M, Ohashi N, Kume T, Yokoi T (2003). Glucuronidation of etoposide in human liver microsomes is specifically catalyzed by UDP-glucuronosyltransferase 1A1. Drug Metab. Dispos..

[14] Gong Q-H (2001). Thirteen UDPglucuronosyltransferase genes are encoded at the human UGT1 gene complex locus. Pharmacogen. Genom..

[15] Mackenzie PI (2005). Nomenclature update for the mammalian UDP glycosyltransferase (UGT) gene superfamily. Pharmacogenet. Genom..

[16] Yueh MF, Bonzo JA, Tukey RH (2005). The role of Ah receptor in induction of human UDP-glucuronosyltransferase 1A1. Methods Enzymol.

[17] Gao S, Bell EC, Zhang Y, Liang D (2021). Racial disparity in drug disposition in the digestive tract. Int. J. Mol. Sci..

[18] Münzel PA, Brück M, Bock KW (1994). Tissue-specific constitutive and inducible expression of rat phenol UDP-glucuronosyltransferase. Biochem. Pharmacol..

[19] Yueh M-F (2003). Involvement of the xenobiotic response element (XRE) in Ah receptor-mediated induction of human UDP-glucuronosyltransferase 1A1. J. Biol. Chem..

[20] Bock KW (2018). From TCDD-mediated toxicity to searches of physiologic AHR functions. Biochem. Pharmacol..

[21] Bock KW (2015). Roles of human UDP-glucuronosyltransferases in clearance and homeostasis of endogenous substrates, and functional implications. Biochem. Pharmacol..

[22] Beutler E, Gelbart T, Demina A (1998). Racial variability in the UDP-glucuronosyltransferase 1 (UGT1A1) promoter: A balanced polymorphism for regulation of bilirubin metabolism?. Proc. Natl. Acad. Sci. U. S. A..

[23] Guillemette C (2003). Pharmacogenomics of human UDP-glucuronosyltransferase enzymes. Pharmacogenom. J..

[24] Huang MJ (2019). Effect of UDP-glucuronosyltransferase 1A1 activity on risk for developing Gilbert's syndrome. Kaohsiung J. Med. Sci..

[25] Consultants, H. & 33, O. Comprehensive assessment of dioxin contamination in Da Nang Airport, Viet Nam: Environmental levels, human exposure and options for mitigating impacts. (Hatfeld Consultants Ltd. North Vancouver, BC, Canada; Ofce 33, Ha Noi, Viet Nam, 2009).

[26] Vuong NB (2023). Association of PKLR gene copy number, expression levels and enzyme activity with 2,3,7,8-TCDD exposure in individuals exposed to Agent Orange/Dioxin in Vietnam. Chemosphere.

[27] EPA, U. S. Tetra- through octa-chlorinated dioxins and furans by isotope dilution HRGC/HRMS. (1994).

[28] Crabtree, G., Son, E., Krokhotin, A., Gourisankar, S. & Chang, C.-Y. ARID1B is a dosage-sensitive regulator of polycomb repressive complex distribution and HOX gene regulation in patient-derived neural progenitors. (2021).

[29] Gardner-Stephen D (2004). Human PXR variants and their differential effects on the regulation of human UDP-glucuronosyltransferase gene expression. Drug Metab. Dispos..

[30] Livak KJ, Schmittgen TD (2001). Analysis of relative gene expression data using real-time quantitative PCR and the 2(-Delta Delta C(T)) Method. Methods.

[31] Guo X (2016). Analysis of uridine diphosphate glucuronosyl transferase 1A1 gene mutations in neonates with unconjugated hyperbilirubinemia. Genet Mol. Res..

[32] Inui H, Itoh T, Yamamoto K, Ikushiro S, Sakaki T (2014). Mammalian cytochrome P450-dependent metabolism of polychlorinated dibenzo-p-dioxins and coplanar polychlorinated biphenyls. Int. J. Mol. Sci..

[33] Hanchard NA (2011). UGT1A1 sequence variants and bilirubin levels in early postnatal life: A quantitative approach. BMC Med. Genet..

[34] Court MH (2013). The UDP-glucuronosyltransferase (UGT) 1A polymorphism c.2042C>G (rs8330) is associated with increased human liver acetaminophen glucuronidation, increased UGT1A exon 5a/5b splice variant mRNA ratio, and decreased risk of unintentional acetaminophen-induced acute liver failure. J. Pharmacol. Exp. Ther..

[35] Yang H (2021). UGT1A1 mutation association with increased bilirubin levels and severity of unconjugated hyperbilirubinemia in ABO incompatible newborns of China. BMC Pediatr..

[36] Guo XH (2016). Analysis of uridine diphosphate glucuronosyl transferase 1A1 gene mutations in neonates with unconjugated hyperbilirubinemia. Genet. Mol. Res..

[37] Han Z, Lin S, Zhong M, Yu D (2020). Correlations of UGT1A1 gene polymorphisms with onset and prognosis of non-small cell lung cancer. Eur. Rev. Med. Pharmacol. Sci..

[38] Zhou P (2014). Association between telomerase reverse transcriptase rs2736100 polymorphism and risk of glioma. J. Surg. Res..

[39] Canu G, Minucci A, Zuppi C, Capoluongo E (2013). Gilbert and Crigler Najjar syndromes: An update of the UDP-glucuronosyltransferase 1A1 (UGT1A1) gene mutation database. Blood Cells Mol. Dis..

[40] Liu X, Cheng D, Kuang Q, Liu G, Xu W (2014). Association of UGT1A1* 28 polymorphisms with irinotecan-induced toxicities in colorectal cancer: a meta-analysis in Caucasians. Pharmacogen. J..

[41] Erlinger S, Arias IM, Dhumeaux D (2014). Inherited disorders of bilirubin transport and conjugation: New insights into molecular mechanisms and consequences. Gastroenterology.

[42] Sugatani J (2013). Function, genetic polymorphism, and transcriptional regulation of human UDP-glucuronosyltransferase (UGT) 1A1. Drug Metab. Pharmacokinet..

[43] Park H (2022). Structure-activity relationships among mono-and dihydroxy flavones as aryl hydrocarbon receptor (AhR) agonists or antagonists in CACO2 cells. Chemico-Biol. Interact..

[44] Xie HQ (2013). AhR-mediated effects of dioxin on neuronal acetylcholinesterase expression in vitro. Environ. Health Perspect..

