# Supplementary material for: Association of UGT1A1 gene variants, expression levels, and enzyme concentrations with 2,3,7,8-TCDD exposure in individuals exposed to Agent Orange/Dioxin
Source: Sci Rep. 2024 Feb 9;14:3315. doi: 10.1038/s41598-024-54004-0 (PMC10853243; doi:10.1038/s41598-024-54004-0)
Supplement: Supplementary file 1 — Supplementary Information. [file 41598_2024_54004_MOESM1_ESM.docx]

**Supplementary data**

**Suppl. table 1. Associations of *UGT1A1* gene polymorphisms rs10929303, rs1042640 and rs8330 with gene expression and enzyme concentrations in Dioxin exposed individuals**

| **Parameters** | | **rs10929303C/T** | | | p |
| --- | --- | --- | --- | --- | --- |
|  |  | *CC (n=72)* | *CT (n=25)* | *TT (n=3)* |  |
| *UGT1A1* gene expression  (2^-ΔCt^) | Median  (25-75%) | 7.47  (2.77-22.26) | 23.75  (6.77-35.02) | 67.18  (35.39-69.59) | 0.02 |
| UGT1A1 enzyme concentration (pg/mL) | Median  (25-75%) | 5690  (4769-6465) | 4547  (3517-6589) | 6547  (6292-6978) | 0.02 |
| **Parameters** | | **rs1042640C/G** | | | P |
|  |  | *CC (n=73)* | *CG (n=23)* | *GG (n=4)* |  |
| *UGT1A1* gene expression  (2^-ΔCt^) | Median  (25-75%) | 7.73  (2.87-23.26) | 23.75  (7.5-38.04) | 35.44  (3.66-69.59) | 0.037 |
| UGT1A1 enzyme concentration (pg/mL) | Median  (25-75%) | 5605  (4776-6436) | 4546  (3510- 6676) | 6292  (5172-6978) | 0.073 |
| **Parameters** | | **rs8330C/G** | | | P |
|  |  | *CC (n=72)* | *CG (n=24)* | *GG (n=4)* |  |
| *UGT1A1* gene expression  (2^-ΔCt^) | Median  (25-75%) | 7.47  (2.77-22.26) | 23.75  (7.5-38.04) | 35.44  (3.66-69.59) | 0.023 |
| UGT1A1 enzyme concentration (pg/mL) | Median  (25-75%) | 5690  (4769-6465) | 4686  (3510-6676) | 6292  (5172-6978) | 0.073 |
| **Parameters** | | ***Haplotype*** | | | P |
|  |  | *CCC (n=168)* | *TGG (n=30)* | |  |
| *UGT1A1* gene expression  (2^-ΔCt^) | Median  (25-75%) | 8.22  (3.24-24.93) | 23.75  (5.06-62.68) | | 0.007 |
| UGT1A1 enzyme concentration (pg/mL) | Median  (25-75%) | 5557  (4723-6495) | 5091  (3547-6763) | | 0.294 |

**Suppl. table 2. Associations of *UGT1A1* gene polymorphisms rs10929303, rs1042640 and rs8330 with gene expression and enzyme concentrations in healthy controls**

| **Parameters** | | **rs10929303C/T** | | | ***p*** |
| --- | --- | --- | --- | --- | --- |
|  |  | *CC (n=72)* | *CT (25)* | *TT (3)* |  |
| *UGT1A1* gene expression  (2^-ΔCt^) | Median  (25-75%) | 4.03  (2.13 - 9.13) | 4.05  (1.83 - 10.27) | 19.8  (2.99 - 48.81) | 0.142 |
| UGT1A1 enzyme concentration (pg/mL) | Median  (25-75%) | 4638  (4225.9 - 5826.4) | 4365.9  (3945.5 - 5168.2) | 4647  (4413.9 - 5600.6) | 0.363 |
| **Parameters** | | **rs1042640C/G** | | | P |
|  |  | *CC* | *GC* | *GG* |  |
| *UGT1A1* gene expression  (2^-ΔCt^) | Median  (25-75%) | 4.03  (2.13 - 9.13) | 4.05  (1.83 - 10.27) | 19.8  (2.99 - 48.81) | 0.142 |
| UGT1A1 ebzyme concentration (pg/mL) | Median  (25-75%) | 4638  (4225.9 – 5826.4) | 4365.9  (3945.5 - 5168.2) | 4647  (4413.9 - 5600.6) | 0.363 |
| **Parameters** | | **rs8330C/G** | | | P |
|  |  | *CC* | *GC* | *GG* |  |
| *UGT1A1* gene expression  (2^-ΔCt^) | Median  (25-75%) | 4.03  (2.13 - 9.13) | 4.05  (1.83 - 10.27) | 19.8  (2.99 - 48.81) | 0.142 |
| UGT1A1 enzyme concentration (pg/mL) | Median  (25-75%) | 4638  (4225.9 – 5826.4) | 4365.9  (3945.5 - 5168.2) | 4647  (4413.9 - 5600.6) | 0.363 |
| **Parameters** | | ***Haplotype*** | | | P |
|  |  | *CCC* | *TGG* | |  |
| *UGT1A1* gene expression  (2^-ΔCt^) | Median  (25-75%) | 4.03  (1.99 - 9.13) | 5.09  (2.38 - 24.93) | | 0.303 |
| UGT1A1 ebzyme concentration (pg/mL) | Median  (25-75%) | 4592.8  (4190.3 - 5626.0) | 4475.1  (4177.9 - 5209.8) | | 0.604 |

**Suppl. figure 1. The correlation between 2,3,7,8-TCDD concentrations and exposure time to Agent Orange/Dioxin.**

The correlations were calculated by using Spearman's rank correlation coefficient. Spearman's rho (r) and *P* values are presented. The left figure presents linear scale for the Y axis while the right figure presents log scale for the Y axis.
